# Soil Bacterial Communities Respond to Climate Changes in a Temperate Steppe

**DOI:** 10.1371/journal.pone.0078616

**Published:** 2013-11-08

**Authors:** Ximei Zhang, Guangming Zhang, Quansheng Chen, Xingguo Han

**Affiliations:** 1 State Key Laboratory of Forest and Soil Ecology, Institute of Applied Ecology, Chinese Academy of Sciences, Shenyang, China; 2 State Key Laboratory of Vegetation and Environmental Change, Institute of Botany, Chinese Academy of Sciences, Beijing, China; Wageningen University, The Netherlands

## Abstract

Climate warming and shifting precipitation regimes are affecting biodiversity and ecosystem functioning. Most studies have focused on the influence of warming and altered precipitation on macro-organisms, whereas the responses of soil microbial communities have been neglected. We studied the changes in the abundance, richness, and composition of the entire bacterial kingdom and 16 dominant bacterial phyla/classes in response to increased precipitation, warming, and their combination, by conducting a 5-year experiment in a steppe ecosystem in Inner Mongolia, China. Watering had a greater effect than warming on almost all the bacterial groups as indicated by changes in all the three attributes (abundance, richness, and composition). The 16 phyla/classes responded differentially to the experimental treatments, with Acidobacteria and Gamma-proteobacteria being the most sensitive. Stepwise regression analyses further revealed that climate changes altered the abundance and richness of bacterial groups primarily through direct routes (e.g., increasing soil water content), and changed the community composition through both direct and indirect routes (e.g., reducing soil total nitrogen content and increasing soil pH). The diverse responses of various bacterial groups could imply some potential shift in their ecosystem functions under climate changes; meanwhile, the indirect routes that are important in altering bacterial composition suggest that specific strategies (e.g., adding NH_4_NO_3_ to maintain soil nitrogen content and pH) could be adopted to maintain soil microbial composition under climate changes.

## Introduction

The rising concentration of atmospheric greenhouse gases has elevated the global mean temperature by 0.76°C since 1850, and the temperature is predicted to continue increasing by 1.8–4.0°C by the end of this century [1]. Precipitation patterns are also expected to shift with climate warming [2], [3]. These climate changes will have profound influences on biodiversity and ecosystem functioning, and subsequently create feedbacks to the climate changes. For example, climate warming reduced plant richness and community coverage in a temperate steppe ecosystem, causing net carbon loss and a positive feedback to climate warming [4], [5]. Previous studies have mainly focused on the response of higher organisms to climate changes. Although soil microbial communities are among the most abundant and diverse groups of organisms on Earth and are responsible for numerous key ecosystem processes such as carbon and nitrogen cycling [6]–[8], their response to climate changes, especially to the combination of climate warming and altered precipitation, has not been comprehensively explored [9].

Less attention has been paid to soil microbial communities primarily because of their extreme complexity and lack of accurate methods to quantify their composition and function. Specifically, they are composed of diverse taxonomic groups (e.g., phyla and classes) with different ecological niches and ecosystem functions. Furthermore, more than 99.9% of them are uncultivable and, hence, we have relatively limited knowledge about their niches and functions [7], [10]–[12]. The traditional PCR-dependent cloning and sequencing technology allows us to acquire tens or hundreds of gene sequences from a soil sample, and thus, is a useful tool for the investigation of some special microbial groups (e.g., the ammonia-oxidizing bacteria [13]) with limited phylogenetic diversity. However, this technology is very labor-intensive and, hence, is not effective for the study of complex microbial groups such as the entire soil bacterial community. Fortunately, the current metagenomic approaches that exploit next-generation sequencing technologies can facilitate investigation of the microbial composition of hundreds of samples simultaneously, accurately, and rapidly [14], [15].

Climate changes may affect soil microbial communities through both direct and indirect routes. We define a direct route as one in which a climate variable (e.g. surface air temperature) alters a synonymous soil physicochemical variable (e.g. soil temperature). Specifically, climate warming may directly affect soil microbial communities by elevating soil temperature, and indirectly affect them by changing other soil physicochemical features such as soil pH, water availability, and carbon and nitrogen content [16], [17]. Similarly, shifting precipitation regime may directly affect soil microbial communities through altering soil water content, and indirectly affect them through changing other physicochemical features. It should be noted that climate warming and precipitation may have interactive effects. Specifically, warming can decrease soil water content, and precipitation can decrease soil temperature. Warming and precipitation may also have interactive effects on soil microbial communities. To gain a mechanistic understanding of the response of soil microbial communities to climate changes, elucidating direct and indirect effects is essential.

The semiarid temperate steppe in northern China is an important part of the Eurasian grassland biome and is sensitive to climate changes [18], [19]. According to the climate history of our study site, the air temperature has increased by 2.4°C over the past several decades (1953–2008), and summer precipitation has also been predicted to increase in the future [1], [20]–[22]. To comprehensively examine the response of soil microbial communities to climate changes in the steppe ecosystem, a field manipulative experiment of watering and warming (mimicking increased precipitation and elevated temperature, respectively) with four treatments (control, watering, warming, and watering plus warming) was conducted since 2005. The pyrosequence technology targeting bacterial 16S rRNA gene was used to measure the structure of soil bacterial communities [14]. The specific questions of this study were as follows: (1) Whether and how will the abundance, richness, and composition of the entire soil bacterial community and the dominant phyla/classes respond to climate warming, increased precipitation, and their combination? (2) What are the relative contributions of the direct and indirect effects to the changes in bacterial abundance, richness, and composition?

## Materials and Methods

### Study Site and Experimental Design

This study was part of a long-term experiment conducted at the Duolun Restoration Ecology Station of Institute of Botany, Chinese Academy of Sciences, approximately 30 km from Duolun County (42°02′N, 116°17′E), Inner Mongolia Autonomous Region of China. This experiment commenced from 2005. As the study site and experimental design have been described earlier [4], [5], [19], we provided only a brief description here. The experimental site was a typical temperate zone characterized by a semiarid continental monsoon climate. Mean annual temperature was 2.1°C with monthly mean temperature ranging from 18.9°C in July to –17.5°C in January. Mean annual precipitation was about 385.5 mm with 80% precipitation occurred from June to September. Soil was chestnut soil (Chinese classification), Calcis-orthic Aridisol in the US Soil Taxonomy classification, with sand, silt, and clay being 62.7%, 20.3%, and 17.0%, respectively. Mean soil bulk density was 1.31 g/cm^3^ and pH was 6.84. This temperate steppe was dominated by perennials, including *Stipa krylovii*, *Artemisia frigida*, *Potentilla acaulis*, *Cleistogenes squarrosa*, *Allium bidentatum*, and *Agropyron cristatum*.

The experiment used a paired, nested design with watering as the primary factor and warming as the secondary factor [4], [19]. There were three pairs of 10 m×15 m plots; one plot in each pair was randomly assigned to watering treatment and the other to the control. At each watering plot, six sprinklers were arranged evenly into two rows with a distance of 5 m between two sprinklers. Each sprinkler covered a circular area with a diameter of 3 m, so the six sprinklers covered the 10 m×15 m plot. In July and August, 15 mm of water was added weekly to the watering plots. Therefore, a total amount of 120 mm water (approximately 30% of mean annual precipitation at the study site) was added each year. Within each 10 m×15 m plot, four 3 m×4 m sub-plots were randomly assigned as the warming and control subplots (each with two replicates). The warming subplots were heated continuously since April 28 of 2005 using 165 cm×15 cm MSR-2420 infrared radiators (Kalglo Electronics, Bethlehem, PA, USA) suspended 2.5 m above the ground. The effect of infrared radiators on soil temperature was spatially uniform within the plot [23]. In the control subplot, one ‘dummy’ heater with the same shape and size as the infrared radiator was suspended 2.5 m high to mimic the shading effect of the heater. Overall, there were six replicates for each treatment (control, watering, warming, and watering plus warming).

### Measurement of Soil Physiochemical Indexes

Soil temperature at the depth of 10 cm was measured with a CR1000 datalogger (Campbell Scientific, Logan, UT, USA) at 1-h intervals from June 4 of 2005. Soil samples were taken on 22 August of 2010. Four soil cores (10 cm deep, 3.5 cm diameter) were collected from each sub-plot (3 m×4 m) at random and thoroughly mixed, part of which was frozen for DNA extraction and the left part was used to measure soil water content, total carbon (TC) content, total nitrogen (TN) content and soil pH. Soil water content was determined as the weight loss after drying for 24 hr at 105°C. Soil TC and TN contents were quantified with the potassium dichromate-vitriol oxidization method and the Kjeldahl acid-digestion method, respectively. Soil pH was measured in 1∶2.5 (*W*/*V*) suspensions of soil in distilled water.

### Measurement of Bacterial Abundance and Community Structure

For each soil sample, we extracted DNA from 0.5 g of mixed soil using the Fast DNA SPIN kit for soil according to the manufacturer’s instructions (Qbiogene, Carlsbad, CA, USA); however, we used 350 µL instead of 50 µL DNA elution solution to elute the DNA in the tenth step of the procedure. The DNA solution was then stored at −20°C until analysis.

The content of bacterial 16S rRNA gene was measured using real-time PCR to represent the abundance of the entire bacterial community [16]. The standard curve was generated using a 10-fold serial dilution of a plasmid containing a copy of the *Escherichia coli* 16S rRNA gene. The 20 µL PCR reaction mixtures contained 10 µL SYBR Premix (TaKaRa Biotechnology Co., Ltd., China), 0.4 µL each of 10 µmol/L forward and reverse primers (Eub338∶5′-ACT CCT ACG GGA GGC AGC AG-3′; Eub518∶5′-ATT ACC GCG GCT GCT GG-3′), 0.4 µL Rox II, 2 µL BSA (10 mg/mL), and 5.8 µL sterile and DNA-free water. The amount of standard and soil DNA samples added per reaction was 1.0 µL (1.2–5.1 ng). The reaction was conducted with a Roche LightCycler™ Real-time PCR system using the following program: 95°C for 1 min followed by 40 cycles of 95°C for 5 sec, 55°C for 15 sec and 72°C for 15 sec. Melting curve and gel electrophoresis analyses were conducted to confirm that the amplified products were of the appropriate size. The bacterial 16S rRNA gene copy number was calculated using a regression equation that related the cycle threshold (Ct) value to the known number of copies in the standards. For each soil sample, the qPCR reactions were repeated three times. We added BSA to the PCR reaction mixtures to reduce the inhibitory effects of co-extracted polyphenolic compounds in the soil. Additionally, three rounds of PCR were conducted after adding known amounts of standard plasmid with the soil DNA extract to estimate the possible inhibitory effects of co-extracted polyphenolic compounds. The inhibitory effects were found to be negligible.

The method of 454 pyrosequences was used to measure the bacterial community structure of each soil sample. The primers 27F (5′-AGA GTT TGA TCC TGG CTC AG-3′) and 338R (5′-TGC TGC CTC CCG TAG GAG T-3′) were used to amplify the fragment of 16S rRNA gene. To measure all 24 samples in a run, a unique 10-mer tag for each sample was added to the 5′-end of the primer 338R [14]. Each 20 µl PCR mixture contained 4 µl FastPfu Buffer (5×; Transgen), 2 µl of 2.5mM dNTPs, 0.4 µl of each primer (5 µM), 0.8 µl of DNA template, and 0.4 µl of FastPfu Polymerase (Transgen). The PCR protocol was as follows: 95°C for 2 min (denature); 25 cycles of 95°C for 30 sec (denature), 55°C for 30 sec (anneal), 72°C for 30 sec (elongate); and 72°C for 5 min (elongate). Three replicates of PCR were performed for each sample, after which the products were combined and purified by agarose gel electrophoresis, recovered, and quantified with PicoGreen using a TBS-380 Mini-Fluorometer. Equal molar concentrations of PCR products for each sample were then pooled and sequenced in a Roche 454 Genome Sequencer FLX Titanium system at Shanghai Majorbio Bio-pharm Technology Co., Ltd. The sequence reads for all samples have been deposited in the National Center for Biotechnology Information Sequence Reads Archive (accession no. SRA057669).

### Data Analysis

The pyrosequence reads were analyzed with the Mothur software [24]. These reads were first assigned to samples according to their tags, after which reads <150 bp in length and with ambiguous characters were removed. The first 150 bp (corresponds to the V3 region of 16S rRNA gene) of the remaining reads were aligned to the Silva database to determine their taxonomic classification and non-bacterial reads were further removed [25]. To minimize the influence of unequal sampling on the following calculated indexes, we randomly selected 3,478 reads for each sample. All these sequences (3,478×24) were clustered into OTUs (operational taxonomic units) with larger than 97% similarity. The OTU number of 3,478 reads for each sample was used to represent the OTU richness of the entire bacterial community. For each pair of samples, the measure of weighted UniFrac was calculated to represent the compositional variation of the entire bacterial community [26]. The Bray-Curtis distance basing on OTU abundance was also calculated to represent the compositional variation of the entire bacterial community. Briefly, we first calculated the difference in the number of reads for each OTU within this pair of samples, and then calculated the sum of the absolute values of these differences for all OTUs. Finally, we divided the sum by 6,956 (3,478×2) to represent the Bray-Curtis distance.

For each of the 16 dominant bacterial phyla/classes, (its sequence number in all 3,478 sequences)/3,478 was calculated to represent its relative abundance. To compare the OTU richness of each phylum/class among different samples, we used a different calculation approach from that for the entire bacterial community, because there was different number of sequences for the same phylum/class among different samples. For each phylum/class, we randomly selected a certain number of representative sequences from a sample and calculated the OTU number represented by these sequences, repeated this process 1,000 times and used the mean OTU number to represent its richness. Although the number of 16S rDNA sequence for some phyla/classes might be very low and the OTU richness could not be estimated quantitatively and accurately from these sequences, this calculation method would still be effective in comparing qualitatively the difference in OTU richness among different samples. For each of the 16 phyla/classes, we also calculated the Bray-Curtis distance basing on the abundance of OTUs to represent the compositional variation between each pair of samples.

Two-way analysis of variance (ANOVA) for a blocked split-plot design was used to determine the main and interactive effects of watering and warming on the abundance and OTU richness of the entire bacterial community as well as the relative abundance and OTU richness of each of the 16 dominant phyla/classes [27]. Before doing two-way ANOVA, the method of Levene test was used to analyse whether these indexes were homogeneous among different treatments and found that all of them were homogenous statistically (*P*>0.05). To visualize the relative difference in community composition among different treatments, we used non-metric multidimensional scaling (NMDS) plots with the software of Primer. Permutational multivariate analysis of variance (PERMANOVA) was further used to reveal the effects of experimental treatments on the composition of the entire bacterial community and that of each of the 16 phyla/classes [28]. For the abundance (or relative abundance), richness, or composition exhibiting significant (*P*<0.05) or marginally significant (*P*<0.10) response to experimental treatments, we used stepwise regression analysis to identify the factor that could effectively explain the changes in the abundance, richness, and composition from the five potential soil physiochemical indexes of soil water content, temperature, TC content, TN content, and pH. Specifically, principal coordinate analyses were first used to determine the difference in community composition among different treatments [29], and the first principal coordinate was subsequently used to represent the dependent variable in the stepwise regression analyses. Two-way ANOVA and stepwise regression analyses were conducted with SPSS software (SPSS 13.0 for WINDOWS).

## Results

### Bacterial Abundance

**Figure 1:**
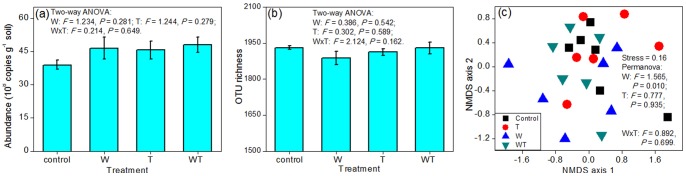
Effects of treatments on the abundance, richness, and composition of the entire soil bacterial community. The effects of block on abundance and richness were non-significant (*P*>0.05), and were not shown in the figure. The compositional variation is represented with the measure of weighted UniFrac, with the r^2^ values between ordination distance and distance in the original space being 0.52 and 0.10 for axis 1 and axis 2, respectively (c). W and T represent watering and warming, respectively. The bars represent one standard error.

**Figure 2:**
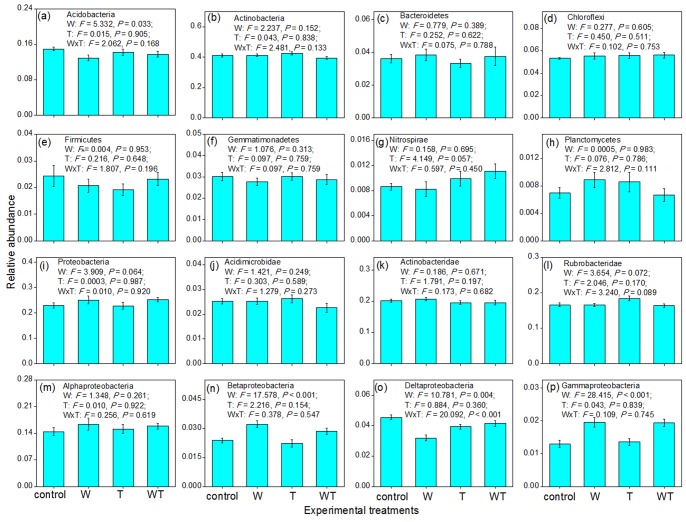
Effects of watering, warming, and their combination on the relative abundance of the 16 bacterial phyla/classes. Two-way ANOVA for a blocked split-plot design was used to test the effects of experimental treatments. The effects of block were non-significant (*P*>0.05), and were not shown in the figure. W and T represent watering and warming, respectively. The bars represent one standard error.

The experimental treatments did not alter the abundance of the entire bacterial community significantly (*P*>0.10; Fig. 1a). However, the treatments significantly (*P*<0.05) or marginally significantly (*P*<0.10; Fig. 2) altered the relative abundance of some phyla/classes. Watering alone decreased the relative abundance of Acidobacteria and Delta-proteobacteria (Fig. 2a, o), but increased the relative abundance of Proteobacteria, Beta- and Gamma-proteobacteria (Fig. 2i, n, p). Warming alone decreased the relative abundance of Delta-proteobacteria (Fig. 2o), but increased the relative abundance of Nitrospirae and Rubrobacteridae (Fig. 2 g, l). Watering and warming had interactive effects. Warming alone increased the relative abundance of Rubrobacteridae, whereas combined warming and watering had a negligible effect (Fig. 2l). Watering or warming alone decreased the relative abundance of Delta-proteobacteria, while the decline in relative abundance was small under concurrent watering and warming (Fig. 2o).

### OTU Richness

**Figure 3:**
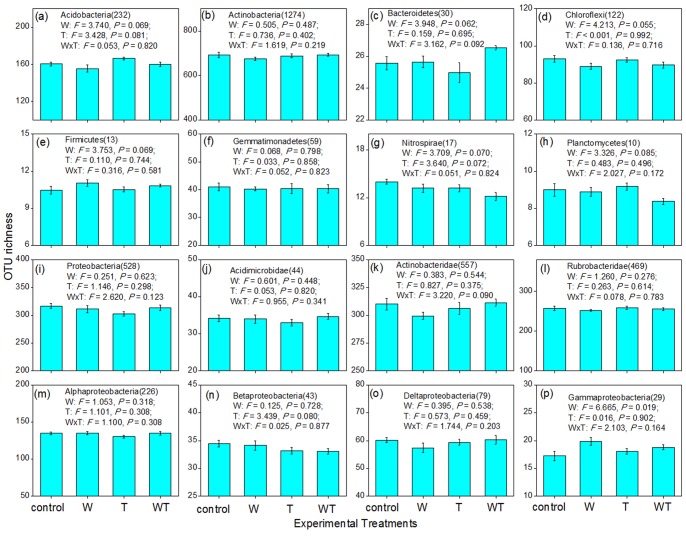
Effects of watering, warming, and their combination on OTU richness of the 16 bacterial phyla/classes. Two-way ANOVA for a blocked split-plot design was used to test the effects of experimental treatments. The effects of block were non-significant (*P*>0.05), and were not shown in the figure. W and T represent watering and warming, respectively. The bars represent one standard error. The number in the brackets following the phylum/class name (e.g., 232 in Acidobacteria(232) in Fig. 3a) represent the sampled sequence number from which OTU richness calculated.

The experimental treatments did not significantly alter the OTU richness of the bacterial community (*P*>0.10; Fig. 1b). However, significant (*P*<0.05) or marginally significant (*P*<0. 10; Fig. 3) changes in OTU richness occurred with some phyla/classes. Watering alone decreased the richness of Acidobacteria, Chloroflexi, Nitrospirae, Planctomycetes, and Actinobacteridae (Fig. 3a, d, g, h, k), but increased the richness of Firmicutes and Gamma-proteobacteria (Fig. 3e, p). Warming alone decreased the OTU richness of Bacteroidetes, Nitrospirae, Actinobacteridae, and Beta-proteobacteria (Fig. 3c, g, k, n), but increased that of Acidobacteria (Fig. 3a). Watering and warming had interactive effects on richness. Whereas warming alone decreased the richness of Bacteroidetes, joint watering and warming increased the OTU richness (Fig. 3c). Watering or warming alone decreased the OTU richness of Actinobacteridae, but the negative effect was negligible when watering and warming occurred concurrently (Fig. 3 k).

### Bacterial Composition

**Figure 4:**
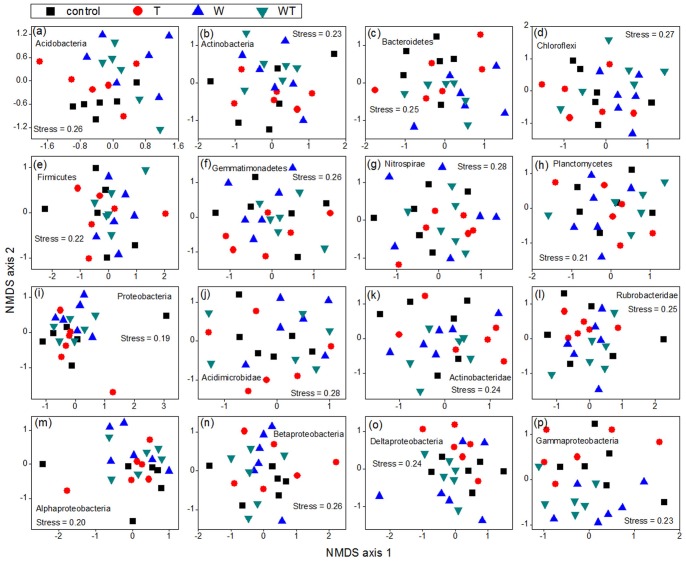
Non-metric multidimensional scaling (NMDS) plots of the 16 bacterial phyla/classes. These plots show the effects of watering (W), warming (T), and their combination on community composition. The compositional variation is represented with Bray-Curtis distance based on the abundance of OTUs.

Watering altered soil bacterial composition significantly (*P*<0.05; Fig. 1c). An NMDS plot of weighted UniFrac values showed that watered plots shifted relative to un-watered plots (Fig. 1c). However, there was a non-significant effect of warming and a non-significant interactive effect of watering and warming on bacterial composition (*P*>0.05; Fig. 1c). When we quantified bacterial compositional variation with the Bray-Curtis distance based on the abundance of OTUs, watering also showed a significant effect (PERMANOVA: watering, *F* = 1.124, *P* = 0.002; warming, *F* = 0.963, *P* = 0.873; watering × warming, *F* = 0.991, *P* = 0.572) and the NMDS plot showed a similar pattern (Figure not shown). Among the 16 dominant phyla/classes, watering only altered the composition of six groups, namely Acidobacteria, Bacteroidetes, Proteobacteria, Acidimicrobidae, Delta-proteobacteria, and Gamma-proteobacteria (*P*<0.10; Fig. 4; Table 1). Warming had non-significant effects and there were non-significant interactions between watering and warming on the composition of the 16 groups (*P*>0.10; Table 1).

**Table 1:** The effects of watering (W), warming (T), and their combination on the composition of the 16 dominant bacterial phyla/classes revealed by PERMANOVA.

	Acidobacteria		Actinobacteria		Bacteroidetes		Chloroflexi	
	*F*	*P*	*F*	*P*	*F*	*P*	*F*	*P*
W	1.390	<0.001*	1.037	0.213	1.134	0.067*	0.961	0.672
T	0.983	0.579	0.975	0.674	0.950	0.717	0.954	0.707
W×T	1.019	0.371	0.995	0.496	1.045	0.283	0.932	0.797
	**Firmicutes**		**Gemmatimonadetes**		**Nitrospirae**		**Planctomycetes**	
	***F***	***P***	***F***	***P***	***F***	***P***	***F***	***P***
W	1.106	0.260	1.084	0.228	0.917	0.631	0.882	0.852
T	0.833	0.835	0.800	0.966	1.175	0.207	0.922	0.744
W×T	0.875	0.767	0.923	0.746	0.882	0.701	0.863	0.893
	**Proteobacteria**		**Acidimicrobidae**		**Actinobacteridae**		**Rubrobacteridae**	
	***F***	***P***	***F***	***P***	***F***	***P***	***F***	***P***
W	1.217	0.004*	1.412	0.003*	1.024	0.342	0.956	0.729
T	0.950	0.786	0.923	0.709	1.059	0.200	0.883	0.963
W×T	0.991	0.530	0.817	0.926	1.047	0.240	0.965	0.682
	**Alpha-proteobacteria**		**Beta-proteobacteria**		**Delta-proteobacteria**		**Gamma-proteobacteria**	
	***F***	***P***	***F***	***P***	***F***	***P***	***F***	***P***
W	1.125	0.102	1.121	0.171	1.253	0.010*	1.725	0.003*
T	0.851	0.961	1.103	0.213	1.080	0.198	1.093	0.301
W×T	0.957	0.649	1.079	0.270	1.086	0.180	0.637	0.979

* denotes *P*<0.10.

### Variables Correlated with Bacterial Abundance, Richness, and Composition

Stepwise regression analyses revealed that the relative abundance of the five groups of Acidobacteria, Rubrobacteridae, Beta-proteobacteria, Delta-proteobacteria, and Gamma-proteobacteria were correlated with soil water content, and the relative abundance of Proteobacteria was correlated with soil pH (Table 2). Although experimental treatments also altered the relative abundance of Nitrospirae (*P*<0.10; Fig. 2g), stepwise regression analyses revealed that none of the five soil physiochemical variables that we examined (water content, temperature, TC content, TN content, and pH) could explain the changes in its relative abundance.

**Table 2:** Variables responsible for the changes in the abundance, richness, and composition of various bacterial groups.

Index	Group	Results	r^2^	*F*	*P*
	Acidobacteria	y = 0.166–0.320 (water content)	0.318	10.278	0.004
	Proteobacteria	y = −0.510+0.103 (soil pH)	0.508	22.706	<0.001
abundance	Rubrobacteridae	y = 0.190–0.248 (water content)	0.197	5.411	0.030
	Betaproteobacteria	y = 0.015+0.136 (water content)	0.441	17.361	<0.001
	Deltaproteobacteria	y = 0.049–0.116 (water content)	0.234	6.730	0.017
	Gammaproteobacteria	y = 0.007+0.117 (water content)	0.567	28.763	<0.001
	Acidobacteria	y = 60.525+6.677 (soil temperature)	0.239	6.902	0.015
richness	Bacteroidetes	y = 24.184+18.237 (water content)	0.192	5.218	0.032
	Gammaproteobacteria	y = 14.768+44.975 (water content)	0.462	18.878	<0.001
	Bacteria	y = 0.878–357.383 (TN content)	0.415	15.612	0.001
		y = 0.914–297.430 (TN content) –2.213 (water content)	0.648	19.349	<0.001
	Acidobacteria	y = 0.326–3.938 (water content)	0.671	44.860	<0.001
composition		y = 1.700–3.130 (water content) –0.199 (soil pH)	0.746	30.838	<0.001
	Bacteroidetes	y = 0.273–3.298 (water content)	0.298	9.330	0.006
	Proteobacteria	y = −2.268+0.313 (soil pH)	0.261	7.757	0.011
	Deltaproteobacteria	y = −2.191+0.146 (soil temperature)	0.233	6.689	0.017

Although experimental treatments altered the OTU richness of nine bacterial groups (*P*<0.10; Fig. 3), stepwise regression analyses revealed that none of the five soil variables could explain the changes in OTU richness of six groups, namely Chloroflexi, Firmicutes, Nitrospirae, Planctomycetes, Actinobacteridae, and Beta-proteobacteria (Table 2). Only the OTU richness of Acidobacteria was correlated with soil temperature, and the richness of Bacteroidetes and Gamma-proteobacteria was correlated with soil water content.

Experimental treatments altered the composition of seven bacterial groups (including the entire bacterial community; *P*<0.10; Figs. 1 and 4; Table 1), but stepwise regression analyses revealed that none of the five soil variables could explain the compositional variation of Acidimicrobidae and Gamma-proteobacteria (Table 2). Variation in composition of the entire bacterial community was correlated with both soil TN content and water content. The composition of Acidobacteria was correlated with both soil water content and soil pH. On the other hand, the composition of Bacteroidetes, Proteobacteria, and Deltaproteobacteria was correlated with soil water content, pH, and temperature, respectively (Table 2).

## Discussion

Although we detected the response of soil microbial communities to experimental treatments at only one time point, the observed results were the 5-year effect of climate changes. In other words, there were accumulative effects. In addition, the response of microbial communities shifted with the intensity of experimental treatments. As the marginally significant response (0.05<*P*<0.10) might become significant (*P*<0.05) with the increasing experimental duration or intensity, we used a lower threshold for significance (*P*<0.10) than the traditional threshold (*P*<0.05) in this study.

Overall, our results demonstrated that watering had a greater effect than warming on soil microbial communities. There were three types of evidence. First, watering significantly altered the composition of the entire bacterial community, whereas warming had no significant effect (Fig. 1c). Second, for each of the three community attributes (abundance, richness and composition), watering affected some of the 16 bacterial phyla/classes, while warming affected only a small number or none of these bacterial groups (Figs. 2, 3 and 4; Table 1). Finally, the abundance and richness of seven bacterial groups were correlated with changes in soil water content, whereas soil temperature was correlated with the abundance and richness of only one group (Table 2). This conclusion is consistent with previous findings that water played a dominant role in the responses of soil/microbial respiration, plant community composition, and ecosystem carbon fluxes to simulated climate changes in this semiarid steppe ecosystem [4], [5], [19]. These phenomena might be due to the fact that this ecosystem was constrained more by water availability than by temperature.

Our results showed that these bacterial phyla/classes responded differently to the experimental treatments in terms of abundance, richness, and composition. In addition, for each bacterial group, its abundance, richness, and composition changed non-synchronously (Figs. 1, 2, 3 and 4; Table 1). For example, while the richness of the Chloroflexi phylum changed significantly in response to watering, its abundance and composition changed non-significantly. On the whole, Acidobacteria and Gamma-proteobacteria were most sensitive among the 16 bacterial phyla/classes, because these two groups exhibited changes in all three community attributes (abundance, richness, and composition) in response to the experimental treatments. Actinobacteria, Gemmatimonadetes, and Alpha-proteobacteria were the most insensitive to experimental treatments, with no apparent changes in abundance, richness, or composition.

Although warming did not change the abundance, OTU richness, and composition of soil bacterial communities (*P*>0.10; Fig. 1), it caused significant reduction in microbial respiration [4]. Furthermore, warming significantly altered soil enzyme activities [27]. These results suggested that climate warming reduced soil respiration primarily through decreasing microbial activities, rather than altering their community structure. In contrast, watering altered microbial respiration [4], bacterial composition (Fig. 1C), and soil enzyme activities simultaneously [27], implying that increased precipitation altered microbial respiration through both shift in their composition and changes in their activities. However, the effect of climate changes on soil microbial activities still needs be investigated in future studies.

Besides soil respiration, soil microbial communities drove many other ecosystem functions, and the changes in their abundance, richness, and composition could guide the potential functional shifts. For example, a large proportion of members in the Chloroflexi phylum could acquire energy and fix CO_2_ through photosynthesis [30]–[32]; thus, the decreased Chloroflexi OTU richness under watering (Fig. 3d) implies that increased precipitation might reduce their carbon sink function. Beta-proteobacteria have been found to play a key role in transforming ammonium into nitrite [13], which is available source of nitrogen to various types of plants. Therefore, increased Beta-proteobacteria abundance under watering (Fig. 2n) suggests that the ammonium-transforming function might be stimulated under increased precipitation. In contrast, decreased Beta-proteobacteria OTU richness under warming (Fig. 3n) implies that this function might be restrained under climate warming. A large proportion of the members of the Nitrospirae phylum could transform nitrite into nitrate [33], [34]; thus, increased Nitrospirae abundance under warming (Fig. 2 g) suggests that this nitrogen-cycling function might be stimulated under climate warming. As there are many plant pathogenic bacteria in the Firmicutes phylum [35], the increased OTU richness under watering (Fig. 3e) implies that the health of plants might be threatened under increased precipitation. Nevertheless, as soil microbial communities are very complex, we still have very limited knowledge about the ecosystem functions of most of the bacterial phyla/classes [36], not to mention the functional shifts under climate changes.

Among the three attributes of all the 17 bacterial groups that we examined, 24 changes in abundance, richness, or composition were at least marginally significant (*P*<0.10; Figs 1, 2, 3 and 4; Table 1), but possible explanatory physicochemical variables–water content, temperature, carbon content, nitrogen content or pH–were identified for only 14 of the changes (Table 2). The absence of correlated variables for the remaining 10 changes can be explained as follows: first, there are other important soil properties (e.g., phosphorus content, trace element contents) that were not measured. Second, although the effects of experimental treatments on the bacterial groups have lasted for 5 years, most of the soil properties were measured only at the time of sampling. A more comprehensive experimental design may reveal additional variables linked to the observed bacterial responses.

For the changes in bacterial abundance or richness that were correlated with a soil physicochemical variable, most of the changes were direct climate effects. For example, the five bacterial groups with changes in relative abundance were correlated with soil water content (direct factor), while only the Proteobacteria were linked to an indirect factor, soil pH (Fig. 2; Table 2). Soil pH was found to be significantly increased by watering treatment in this steppe ecosystem (unpublished data). Similarly, the three bacterial groups with changes in richness were correlated with the direct factors, soil water content or soil temperature (Fig. 3; Table 2). In contrast, changes in bacterial group composition that were correlated with soil physicochemical factors were linked to both direct and indirect factors. Specifically, the compositional variation of the entire bacterial community was explained by both water content (direct factor) and TN content (indirect factor), and that of Acidobacteria was explained by both water content (direct factor) and soil pH (indirect factor; Table 2). Furthermore, the compositional variation of Bacteroidetes and Proteobacteria were explained by water content (direct factor) and soil pH (indirect factor), respectively (Table 2). Although the composition of Deltaproteobacteria community changed in response to watering (Table 1), it was linked to the indirect factor, soil temperature (Table 2). Overall, climate changes not only altered the abundance and richness of soil bacterial communities primarily through direct routes, but also altered their composition through both direct and indirect routes. Abundance and richness of a bacterial group are primarily determined by the environmental capacity (e.g., the total amount of energy), and hence, the direct effects of watering (soil water content) and warming (soil temperature) are logical. In contrast, besides environmental capacity, the composition of a bacterial group is also partly determined by environmental heterogeneity, and thus, watering/warming will also affect bacterial composition through alteration of indirect soil properties [16]. For example, under increased watering, the nearly neutral soil became alkaline; meanwhile, stimulated growth of plant community in this semiarid steppe transformed most of the soil nitrogen to aboveground plant biomass, resulting in a reduction in soil TN content (unpublished data). Therefore, to maintain soil bacterial composition under climate changes, management strategies may be required to address the changes in the indirect factors such as soil TN content and pH, in addition to alterations in the direct factors.

Although climate warming had small influence on the abundance, richness and composition of the entire soil bacterial community in the semiarid temperate steppe, it was found to have much larger effect in a tall grass prairie in central Oklahoma [6], [9]. This difference is due to two potential causes. First, the experimental intensity and duration are different. In other words, climate warming may have larger influence with the increasing experimental duration or intensity. Second, water/precipitation is found to be important in affecting soil bacterial communities in both ecosystems [9], and the mean annual precipitation in the steppe (∼386 mm) is smaller than that in the prairie (∼900 mm). We propose that climate warming primarily affects soil bacterial community through decreasing soil water content (drying the soil), and that the bacterial communities in the semiarid steppe are more adaptive to the dried soil environment than those in the prairie due to the long-term evolutionary process. Thus, soil bacterial communities in the steppe are more insensitive to climate warming. However, these hypotheses need further test. Anyway, the effect of precipitation/water should be taken into account with care in studying the influence of climate warming on soil microbial communities.
